# Trust-Based Smart Contract for Automated Agent to Agent Communication

**DOI:** 10.1155/2022/5136865

**Published:** 2022-09-17

**Authors:** Halima Mhamdi, Ben Othman Soufiene, Ahmed Zouinkhi, Obaid Ali, Hedi Sakli

**Affiliations:** ^1^MACS Research Laboratory, National Engineering School of Gabés, Gabés University, Gabes 6029, Tunisia; ^2^PRINCE Laboratory Research, ISITcom, Hammam Sousse, University of Sousse, Sousse, Tunisia; ^3^Ibb University, Department of Computer Science & Information Technology, Tudun Wada, Yemen; ^4^EITA Consulting 5 Rue Du Chant des Oiseaux, 78360 Montesson, Montesson, France

## Abstract

Blockchain technology is now regarded as one of the most interesting and possibly innovative technologies. It enables information to be stored and exchanged securely and transparently without the need for a centralized authority to regulate it. Some of the primary benefits of this technology are the atomicity of the stored data. Given its features, this technology has the potential to provide answers to challenges encountered in a very sensitive sector, namely, Internet of Vehicles (IoV). In IoV, vehicles and service providers autonomously capture and produce data without human intervention. This exchanged data must meet certain criteria such as decentralization, automation, security, and stakeholder trust management. To overcome these challenges, the integration of blockchain technology and multi-agent systems is a key solution. Based on smart contracts, the proposed solution consists of exploiting role-based access control (RBAC) and attribute-based access control (ABAC) techniques. This solution removes the central authority (CA) to reduce maintenance costs and eliminate legacy threats from centralized systems. The results, obtained from consumption costs, show that the developed platform is characterized by security, availability, and privacy.

## 1. Introduction

With its evolution, the Internet of Things (IoT) is included in various sectors: healthcare, agriculture, energy, industry, and intelligent transportation systems [1]. It allows communication between different types of objects at any time and in real-time. In 2021, the number of connected objects was estimated at 35.82 billion and will exceed 75.44 billion by 2025 [2]. Included in Vehicular Ad-Hoc Networks (VANET), the vehicle becomes the connected object par excellence, forming a new paradigm called the Internet of Vehicles (IoV). In the IoV, vehicles become capable of establishing interactions with themselves and with other connected surrounding objects. This interaction is ensured thanks to the permanent Internet connectivity offered by the IoV. The vehicles will enable consumers to become familiar with the Internet of Things through new services that bring more comfort, precision, and efficiency. Indeed, road safety and traffic fluidity as existing applications can be improved by this new concept. Other new services such as vehicle maintenance, parking access, fuel stations, and tolls are developed. As a result, vehicles and service providers autonomously capture and generate data without human intervention [3]. This exchanged data must meet certain criteria. The high connectivity of vehicles and connected objects in the IoV network facilitates malicious attacks. Therefore, the different parties involved in the communication process must interact in a secure and confidential manner. Let us also not forget that the centralized architecture of intelligent vehicle communication models can lead to an interruption of the IoV network if the central server fails. Therefore, decentralization of the system is a very important challenge. To overcome these challenges of decentralization, automation, security, and stakeholder trust management in IoV, the fusion of blockchain technology and multi-agent system is a key solution.

Distributed ledger technology is often known as blockchain technology. In this peer-to-peer network, data is manipulated in an immutable, decentralized, and confidential manner. These characteristics increase the demand for the use of blockchain technology in various sectors. Included in the supply chain, the blockchain is involved in terms of data security and traceability [4]. Ethereum blockchain and the ERC20 interface are the basis of the proposed system. This system allows automating P2P exchanges, reducing the cost for a trusted third party by ensuring interoperability. Therefore, it guarantees the reliability and trust of information access. Combined with IoT, an extensive study exposed by Saxena et al. [5] and Bhushan et al. [6]. The developed IoT applications are characterized by privacy, confidentiality, and security of managed data. The authors in their article [7], with the same objective of ensuring security and privacy, have outlined the contribution of this technology in the design and development of the smart city. The integration of blockchain technology in IoV and health is the subject of several review articles [8–10].

Any real or abstract entity that can communicate with other entities in its environment is defined as an agent. The set of autonomous agents thus forms a multi-agent system (MAS). The collaboration between the components of the system allows for solving a predefined problem [11]. The MAS allows automating the interactions between the agents forming a fully decentralized system. Indeed, the MAS consolidate the efficiency and the confidence of the human/machine or machine/machine communication. It also ensures the security of the agents. The authors [12] have exploited the multi-agent system and propose a trust management system between agents by using the technique of identification of agents via private and public keys. In the same context, ensuring security and trust in the multi-agent system shows relevant and reliable results in energy exploitation in a smart home, in logistics, and in an industry [13, 14].

Several researchers have combined the multi-agent system with blockchain technology to take advantage of its security and privacy features. Among the applications developed are carpooling systems, energy consumption, as well as food supply chain monitoring, and electronic voting [15].

Applied in the IoV, our proposed solution consists in designing a decentralized communication system between vehicles and vehicular service providers such as automatic fueling stations using both technologies: multi-agent system and blockchain. The main objective is to ensure security and trust between the agents of the system by establishing automated communication without human intervention via smart contracts. The focal contributions of this work are summarized as follows:

1. We propose an autonomous agent-to-agent communication system.
2. To reduce operating and maintenance cost and eliminate legacy threats from a centralized system, we combine the MAS technology with the blockchain technology.
3. We propose a smart contract-based distributed and dynamic access control mechanism that enables system agents to share data securely.
4. We implement our scheme on Ethereum blockchain. The performance evaluation, experimental results, and security analysis clearly prove the systems' efficient.

The rest of this paper is organized as follows: The second section is devoted to the study of existing works regarding the application of blockchain technology in IoV. The proposed system architecture is introduced in detail in Section 3. While Section 4 describes the simulation platform and the obtained results. The analysis and performance of the system is included in Section 5. Finally, Section 6 concludes this paper and gives some hints for further research.

## 2. Related Works

The IoV concept is based on vehicular communication. Vehicles are thus intelligent objects that share and exchange information with each other and/or with other connected objects. Vehicle-to-object (V2X) communication enables the creation of a multitude services for the intelligent transport ecosystem [16]. In V2X, security and trust are two essential elements because the manipulated data can be subject to attacks. To overcome this problem, many scientists have recently become interested in using blockchain technology in vehicular networks. Its integration with data trading makes it possible to facilitate P2P exchanges.  Table 1 summarizes the several works presented in this section.

In this context, Chen et al. [17] proposed a framework based on a consortium blockchain that allows the exchange of data that is both secure and efficient between buyers and sellers. The developed solution is an iterative double auction method. It aims to maximize the welfare of the society while preserving the privacy of buyers and sellers so that more users are encouraged to take part in data trading. In addition, the cost of data transmission is also considered to improve the stability of the system. This system is composed of 3 entities, namely, vehicles, edge layer, and blockchain layer. Vehicles interact with each other to exchange data. To access specific types of data, any car in the system must pay a virtual token. Depending on its current state and data needs, the purpose of each node may change. Data brokers are edge servers in the edge layer of the IoV framework. Each car communicates its data needs to the nearest data broker, which then informs local providers. Blockchain, smart contracts, and miners are the three main mechanisms in the blockchain layer. The blockchain is used to ensure high credibility and security, smart contracts allow for a variety of user-designed algorithms, and mining ads to the resilience of the system. The edge layer and the blockchain layer can work together in two ways. For ledger storage and consensus, the edge layer provides significant storage and computational resources. To create trust and ensure security, the blockchain layer enables an edge layer.

Using blockchain technology, the authors in [18] offer a secure and decentralized IoV exchange system. This system uses an incentive-based investment and pricing structure, enabling car lending. By promising to pay interest and an incentive, a vehicle with a loan request can borrow from a group of cars. To optimize the payoffs for borrowers and lenders, a two-stage Stackelberg game is established. The perfect subgame equilibrium at each stage is analyzed by inverse induction. Furthermore, for secure data exchange and peer-to-peer financing services, the authors exploited the consortium blockchain, smart contracts as well as the proof of work as a consensus mechanism. The numerical results show that both the suggested independent and uniform pricing systems are effective.

Yang et al. [19] develop a blockchain-based decentralized trust management approach in vehicle networks. The Bayesian inference model is used by cars in this system to verify the signals received from nearby vehicles. They use the combination of proof-of-work (PoW) and proof-of-stake (PoS) consensus method. Through this fusion, all RSUs work together to keep the trust blockchain up to date, reliable, and consistent.

The study in [20] proposes a vehicle-to-vehicle (V2V) energy exchange mechanism to solve the driving resistance problem of electric vehicles. In the distributed BIoV with imperfect information sharing, Bayesian pricing is used. The proposed approach has several advantages over a static set with all available data. Electricity transactions are more trustworthy, secure, and reliable using blockchain technology. For starters, the public/private key pair and digital signature are used with blockchain to address the security concerns of vehicle users. Second, the pricing method is incorporated into the smart contract for electric transactions, which not only reduces the dependency on the middleman, but also ensures that the transaction is fair.

Javaid et al. [21] address the problem of trust in the IoV network. They rely on blockchain technology to overcome this challenge by developing a trust protocol. The proposed solution uses both smart contracts and dynamic proof of work (dPoW) as consensus algorithm as well as certificates and nonclonable physical functions. These 4 mechanisms work together to ensure security and trust in vehicle registration. The resulting solution is characterized by throughput scalability, decentralization, low latency, and vehicle privacy protection. Similarly, to solve the problem of security during the transmission of messages between vehicles in the IoV network, a protocol named IoV-SMAP is implemented. This protocol makes it possible to overcome security flaws while guaranteeing the confidentiality and authentication mechanisms of the users.

In their paper [22], the authors propose a protocol for vehicle accident detection and notification in an ITS environment using a new authentication method called BCAS-VADN. They use blockchain technology to store the information generated by this method. In addition, BCAS-VADN offers end-to-end encryption and the ability to store a cryptographic hash signature of the interactions included in the blocks of the blockchain, thus providing a method of validating the integrity of transactions. The proposed system is composed of 4 phases: registration of all entities in the system, their authentication, verification, and addition to the blockchain and the dynamic addition of new nodes to the network. The use of blockchain technology and the Practical Byzantine FaultTolerance consensus mechanism reinforce the security since it is impossible to delete or modify the data exchanged in the network.

PSEV [23] is a framework based on blockchain technology that allows the creation of payments and secure communications between the different participants of the IoV system. This solution ensures the automation of data exchange processes using smart contracts. To validate the proposed solution, the authors use the Ethereum blockchain to develop a decentralized real-time parking reservation and payment application. The designed system is characterized by a low cost and a reduced execution time compared to existing systems. It also ensures integrity, immutability, and confidentiality.

Singh et al. [24] proposed a mechanism for trust management in the IoV system. The studied approach is to overcome the scalability problem by exploiting blockchain technology. The decentralized feature is ensured by using smart contracts and Proof of Work as a consensus mechanism. For validation, the authors resort to the use of the Ethereum blockchain platform that allows examining the performance of the system considering the average throughput and execution time. For the same purpose, Zhao et al. [25] also addressed the problem of trust management in the IoV system. They developed a protocol that bundles the practical Byzantine fault-tolerance consensus mechanism with the proof-of-work. This linkage ensures the anonymity of vehicles via identity-based group signature.

## 3. System Design and Architecture

The proposed system is a platform allowing the communication between autonomous agents by integrating the blockchain technology and the multi-agent system (MAS). The architecture of the system is composed of 2 main parts: a MAS that allows communication between agents and a public blockchain network (BC) that allows to store all transactions and smart contracts.

Blockchain technology, namely, smart contracts, allows agents to engage more quickly, automate business procedures, and transmit orders across them. The data generated by multi-agent modeling can be kept in the blockchain. Agents are critical in IoV systems, and with blockchain, they may provide even more value to the ecosystem, increase its dependability, and facilitate its expansion.

### 3.1. System Design

**Figure 1:**
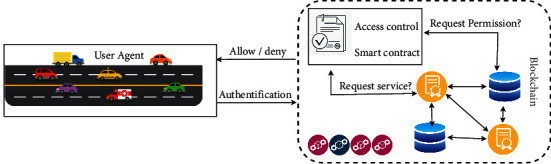
Proposed system model.

Our system's model is depicted in Figure 1. Connected automobiles can communicate with a variety of items. They are also linked to the Internet through the provision of intelligent services. The most important feature is the decentralized exchange of information between different entities. The proposed model is composed of two actors or two agents: service providers and vehicles. All agents have their blockchain address. The blockchain records the transactions between the actors, as well as information about the vehicle and services. The smart contract ensures these transactions automatically validated by consensus mechanisms. The proposed model is based on 3 main concepts as follows:

#### 3.1.1. Smart Contract

Smart contracts are the most important component of any blockchain framework as they fulfill basic functions. For the design of our framework, the first step is the deployment of different smart contracts either for system stakeholder's enrollment or for authentication to benefit from a service and pay the provider.

#### 3.1.2. Authentication

Access to the system requires user authentication through Ethereum addresses for each agent. After authentication, the vehicle agent can consult and communicate with the service provider agent.

#### 3.1.3. Access Control

Access control is a process that allows only authorized entities to manage information and controls this information. In our case, to use a service, the vehicle agent sends an access request via the smart contract that verifies the identity of the requester and authorizes him to send a request to the service provider.

### 3.2. Blockchain and Agent Communication Process

The communication process between our system's agents is divided into five stages: agent enrollment, agent registration, agent authentication, request service, and payment. The registration and communication process between agents based on the smart contract is explained by Algorithms 1–3 as described below. For convenient reference, Table 2 lists the various symbols and acronyms used in this subject.

#### 3.2.1. Agent Enrollment

In this step, the system administrator assigns to each Ethereum address an agent type or a role. This designation is recorded in a hash table via a smart contract. The added role is then used when adding an agent or in the handling of transactions in the communication process. However, the deployment of different smart contracts required by our system was the object of this step.

#### 3.2.2. Agent Registration

The registration step consists of adding the agents to the system. After verifying the account address, the agent can add its information to the Agent_DB through a smart contract. In this phase, the smart contract saves the characteristics or attributes of each agent, especially the identifier and its account in token. Upon successful registration, agents are allowed to join the blockchain.

#### 3.2.3. Agent Authentication

To communicate and benefit from a service, the registered agents authenticate themselves. Two types of access control are used: Role-Based Access Control and Attribute-Based Access Control. The use of the “msg.sender” variable of the OpenZeppelin library allows for identifying and validating the agent's address. On the other hand, during the payment phase, it is necessary to control the access to the data of the agents such as the balance account.

#### 3.2.4. Request Service

Once enrolled, the vehicle agent sends a request to the system to consult the list of available service providers. Then it chooses the appropriate provider. In this step, a smart contract is established between the stakeholders. This contract contains all the necessary criteria to use a service such as the minimum amount that must be in the applicant's portfolio.

#### 3.2.5. Payment

After having exploited the desired service, a request is sent to the blockchain to complete the payment phase. The request containing the amount to be paid is managed automatically by a smart contract. This smart contract accesses the account of the stakeholder in this process and settles what is due.

### 3.3. Smart Contract Design

The suggested smart contract design comprises of three contracts: Registration_Contract, VA_SA_Contract, and Access_Contract.

#### 3.3.1. Registration_Contract

It is designed to add Vehicle_Agent and Service_Agent in the system. The information provided by each agent is then stored in Vehicle_Agent_DB and Service_Agent_DB. These two registration tables represent a distributed database containing all registered agents. The authentication and authorization of agents are also controlled by the logging database.

#### 3.3.2. VA_SA_Contract

It is a binding agreement between the vehicle agent and the service agent. It includes the system's stakeholder ID as well as the service charge.

#### 3.3.3. Access_Contract

It maintains access control among system agents. When sending a service request from the vehicle agent to the service agent, this contract controls the access of this request. This contract is also involved in the payment phase. When access to the service is finished, using ABAC, it deposits the requested amount from the vehicle wallet to the service wallet.

## 4. System Implementation and Analysis

**Figure 2:**
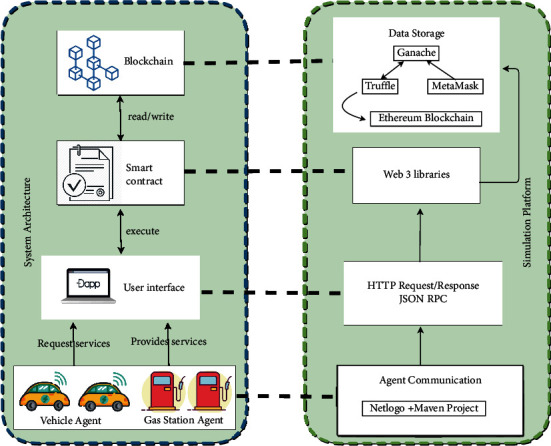
Proposed system diagram.

Figure 2 presents the system operation as well as the simulation tools used, which will be discussed later. The proposed platform consists of two agents: VA and GSA. The latter two form an autonomous system allowing the automation of “M2M communication” processes. First, VA, when it reaches a fuel threshold, will choose a GSA from a list of stations registered in the blockchain. This list provides information about the location of the station and its rates and its availability. The loading process requires the authentication of the vehicle requesting the service and the provider station. This is done via decentralized application interface (Dapp). BC is used to eliminate the middleman for authentication, payment, and automation of the service via smart contracts. After choosing the station, a smart contract is established between the two parties for the loading. The vehicle pays the loading amount which depends on the loaded quantity, type, and price, but the money is kept in the blockchain. When the loading process is completed, the smart contract automatically pays the agreed amount of money to the station account.

### 4.1. Simulation Platform

The agents of our system are modeled by the Netlogo Framework through which two types of communication are implemented between the different agents: the request and the reception of a transaction. The conversation between the agents is ensured by an agent communication language (ACL). To facilitate the communication between Netlogo, the Dapps, and the blockchain network, the Maven extension is used.

**Figure 3:**
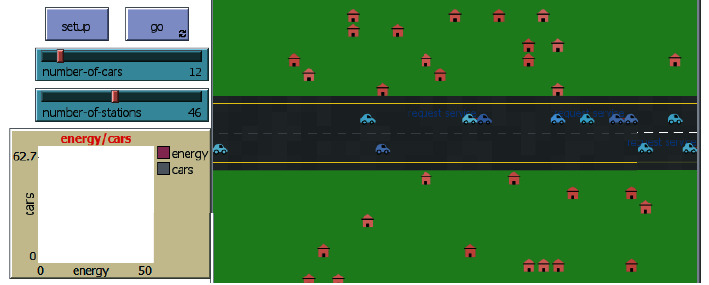
Netlogo interface.

Each agent has an account in the Ethereum blockchain. To access this account, we use the web3.js library through an HTTP connection in JSON RPC format. The truffle development environment is used to compile and migrate smart contracts to the blockchain. Metamask is used to implement Ethereum wallet functions that allow participants to control the Ethereum account information and make the transaction payment. Any changes submitted to the transactions are recorded on the blockchain network (see Figure 3).

Table 3 provides a list of the technologies used to implement the system and a brief description of each.

### 4.2. Results and Performance Evaluation

In this section, we will present the results of smart contracts deployment of our system as well as the consumption cost and transaction cost.

#### 4.2.1. Smart Contracts Deployment

**Figure 4:**
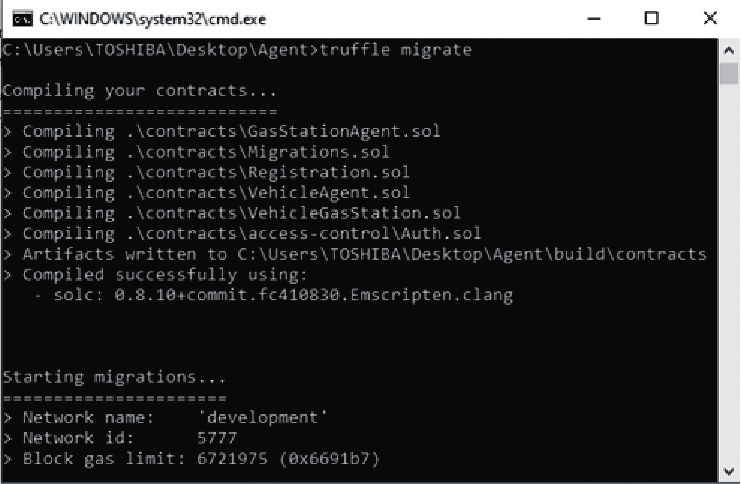
Smart contracts compilation.

Figure 4 depict the compilation and migration of smart contracts to the blockchain ganache. Five smart contracts are compiled and deployed in the “development” blockchain: “GasStationAgent.sol,” “Registration.sol,” “Auth.sol,” “VehicleAgent.sol,” and “VehicleGasStation.sol.” The entire cost of this transaction as shown by the migration result, is 0.003796388 ether, which is the equivalent of £ 13.21. This conversion is done on March 28th, 2022.

**Figure 5:**
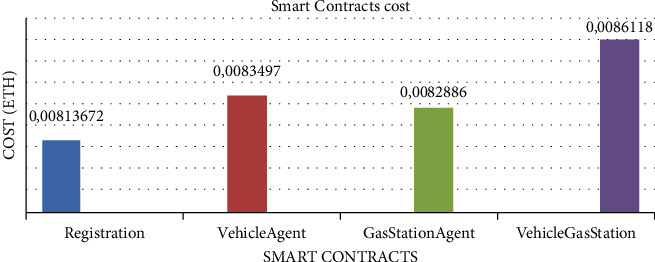
Smart contracts cost.

Figure 5 represents the execution costs of the different smart contracts in our system. We notice that the cost of the vehicle gas station contract is higher than the other contracts. This contract contains the main functions of the system, such as communication between the different agents as well as the access control functions ABAC and RBAC. While registration contract consumes less gas since it just assigns a role to the agents in a table containing the accounts and roles. Vehicle agent and gas station agent contracts take care of adding the relevant information to each agent. Their gas consumption costs are nearby.

**Figure 6:**
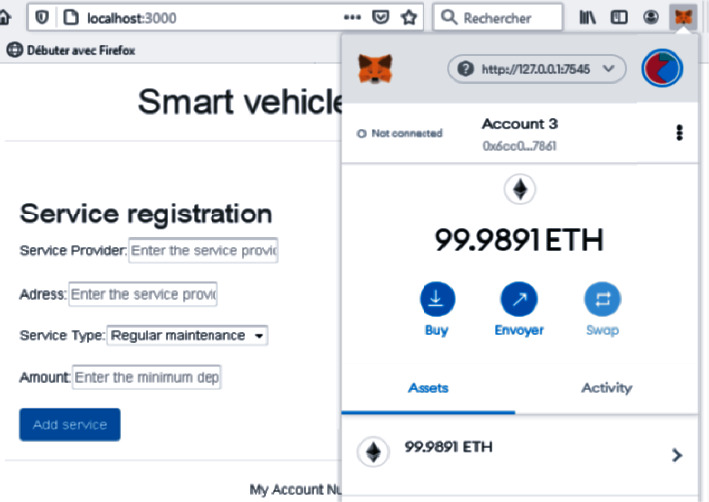
Vehicle registration data screen.

Following the transfer of our smart contract, we will build a local virtual server containing the client-side application using the truffle framework. To join to our blockchain network, we need to connect to our Metamask portfolio as shown in Figure 6.

#### 4.2.2. Consumption and Transaction Cost

The system's cost of consumption is calculated in terms of the number of gas units utilized by smart contracts. Gas is a unit of measurement that estimates the processing power required for transaction execution. Gas units are transformed into ether value in Ethereum. To calculate the gas fees for each transaction in our system, we apply the following equation:

(1) $$\begin{array}{c} \text{Transaction Fee}=\text{gasUsed}\times \text{gasPrice}. \end{array}$$

where gas used is defined depending on the storage and processing quantity for each transaction and gas price denotes the amount of Gwei necessary for the transaction. Let us take the example of the agent enrolment function which allows assigning to each agent a role and account. The gas used of this is 112883 and gas price is 20 Gwei. So,

(2) $$\begin{array}{c} \text{Transaction Fee}=112883\times 20=2257660\text{ Gwei}=0.000225766\text{ Ether}. \end{array}$$

We have calculated the transaction costs for the different smart contracts.  Table 4 shows the gas consumed. Note that the transaction cost for reading data from the blockchain, such as the “getVehicleAgent,” “getAllGasStation” and “readMessage” functions, is null. Since mining is not required while getting messages from blocks, and no changes are required for the smart contract, this function does not incur any extra costs.

**Figure 7:**
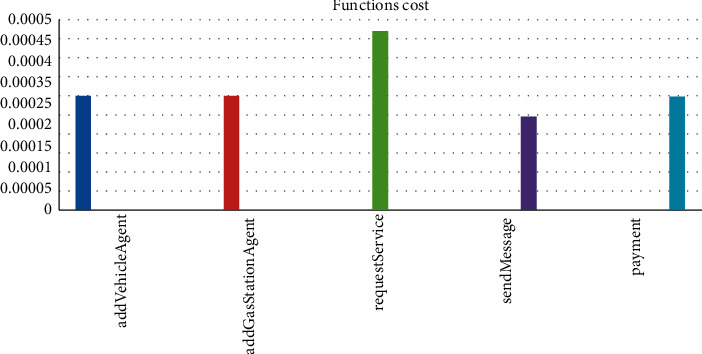
Functions cost.

Figure 7 shows the gas consumed by each function in our system. The functions of the agent communication subsystem consume more gas with 0.000470134 Eth, 0.000297561 Eth and 0.000244863 Eth for the respective requestService, payment, and sendMessage functions. This high cost is due to the operations and the data they manipulate. On the other hand, the functions that manage the addition of agents: addVehicleAgent and addGasStationAgent consume about 0.0003.

### 4.3. Comparison

Table 5 compares the proposed method to other works in the field in terms of performance parameters and used technologies. As previously stated, we were unable to locate a paper that shared our concerns. As a result, several works implemented a solution based on blockchain technology and smart contracts, but they did not use multi-agents' system. It is important to note that in the related works, we only focused on papers that are more relevant to our use-case and chose from them; as a result, some papers are not related to alternative access control methods.

#### 4.3.1. Security

The use of the RBAC and ABAC mechanisms ensures the security of our proposed framework. So, no third party is allowed to access the system. Let us not forget also that blockchain is protected with mechanisms and protocols. Therefore, agent data can be handled reliably and confidentially. Only trustworthy persons have access to these data. The system denies access to any untrusted third party attempting to access the system.

#### 4.3.2. Availability

The use of the public blockchain allows the system to be accessible to anyone who need a gas station service.

#### 4.3.3. Trustfulness

Trust is maintained with access control via user registration as well as restricting access to the data of our system stakeholders.

## 5. Conclusions

In this study, we propose a smart contract-based distributed and dynamic access control mechanism that ensures an autonomous agent-to-agent communication system. As a result, blockchain technology is combined with multi-agent systems and access control in order to guarantee security and trustfulness. To ensure that these features are implemented, various smart contracts are strategically placed. Vehicle Agent and Gas Station Agent are dedicated to the management of the agents' data. The smart contract vehicle Gas Station allows the exchange of data between vehicles and service providers and payment. The search for a neighboring service is handled by the multi-agent system. To evaluate the performance of the proposed system, the calculation of the costs of the different smart contracts and the costs of the functions that they contain is performed. The results obtained show that the developed platform is characterized by security, availability, and privacy.

In the proposed system, several data are manipulated and need to be stored securely so their storage is a challenge to consider when implementing a real system. So as a perspective cloud storage is considered. In this case, the data of the different agents as well as the smart contracts are stored in the cloud and the blockchain just contains their URL. Another perspective is the implementation of an access control mechanism for the access to the cloud.

## Figures and Tables

**Algorithm 1:**
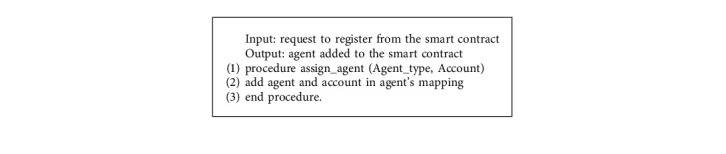
Agent enrollment.

**Algorithm 2:**
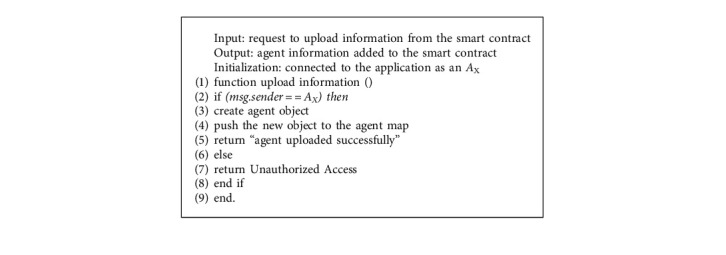
Upload agent information

**Algorithm 3:**
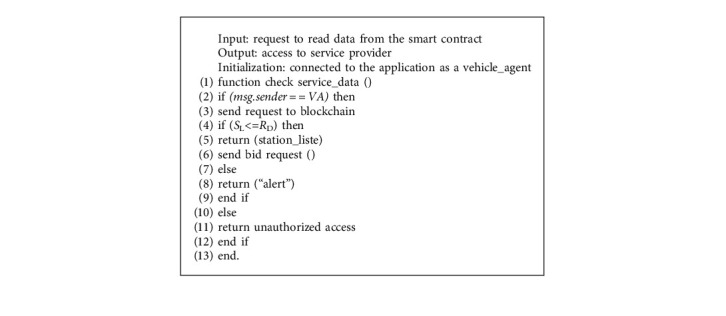
Search service provider.

**Table 1:** Summarized literature review.

References	Objectives	Blockchain			Performance		
		Type	Consensus algorithm	Smart contracts	Security	Privacy	Trust
[17]	Ensuring secure and efficient data exchange	Consortium blockchain	PoW	^*∗*^	^*∗*^		
[18]	To facilitate efficient IoV data-trading	Consortium blockchain	PoW	^*∗*^	^*∗*^	^*∗*^	
[19]	Ensure trust and decentralized of incoming communications between vehicles and their neighbors	NM	PoW and PoS				^*∗*^
[20]	Solve the driving endurance problem of an electric car by implementing a vehicle-to-vehicle energy exchange system	Fabric blockchain	PoA	^*∗*^	^*∗*^		
[21]	Ensure security and confidence in vehicle registration	Ethereum blockchain	dPoW	^*∗*^			^*∗*^
[22]	Provide security when transmitting messages between vehicles in the IoV network	NM	NM		^*∗*^		
[23]	Detection and notification of vehicle accidents in an ITS environment	NM	PBFT		^*∗*^		
[24]	Provide automation of data exchange processes in a secure way between the different participants of the IoV system	Ethereum blockchain	NM	^*∗*^	^*∗*^		
[25]	Overcome the scalability problem for trust management in the IoV system	Ethereum blockchain	PoW	^*∗*^			^*∗*^
This paper	Ensure security and trust between the agents of the system by establishing automated communication via smart contracts.	Ethereum blockchain	PoW and PoS	^*∗*^	^*∗*^	^*∗*^	^*∗*^

**Table 2:** Symbols and used acronyms.

Symbol	Description
*A*_X_	Stakeholders agent in the communication process
VA	Vehicle agent
GSA	Gas station agent
*S*_L_	Station localization
*R*_D_	Remaining distance
M2M	Machine-to-machine

**Table 3:** Technologies used for the realization of the system.

Technologies		Description
Blockchain	Ganache	A personal blockchain for local development. It allows to deploy smart contracts, develop applications, and run tests. Ganache provides 10 Ethereum accounts with a balance of 100 ether for each account, as well as a graphical interface to examine everything that happens in this blockchain.
	Truffle	A framework that provides a suite of tools for developing Ethereum smart contracts and a client-side interface, testing and deploying smart contracts in any Ethereum network.
	Ethereum	A distributed computing platform based on a public blockchain that can support advanced custom smart contracts using the turing-complete programming language.
	Smart contract: solidity	An object-oriented programming language dedicated to writing smart contracts. It is used to implement smart contracts on various blockchains, including Ethereum

Web server	Meta mask	A plugin allows to transform a web browser into a blockchain browser. It also allows the management of blockchain accounts, as well as ether funds to pay transactions
	Web3.js	A collection of libraries allowing interaction with a local or remote Ethereum node, using an HTTP or IPC connection in JSON RPC format.

Multi-agent system	Netlogo	A well-known and widely used cross-platform modeling and simulation environment for complex systems of simultaneously interacting agents, written on top of the Java virtual machine. Netlogo offers a wide range of generic features and operators to its users. In addition, to compensate for missing functionality, Netlogo is compatible with other platforms and libraries. Conversely, Netlogo can also be called and controlled by other programs. For this purpose, Netlogo provides a Java API.
	Maven extension	A program construction management system developed by the Apache foundation. It is based on the definition and use of POM (project object model) files containing all the instructions guiding the correct construction of the program.

**Table 4:** Function cost.

System	Function	Transaction cost (eth)	Price ($)
	Deploy contracts	0.003796388	13.21
	Agent enrolment	0.000225766	0.79

Vehicles agent management	addVehicleAgent	0.000332948	1.16
	getVehicleAgent	0	0
	getAllVehicleAgent	0	0

Gas stations management	addGasStationAgent	0.000301534	1.05
	getGasStationAgent	0	0
	getAllGasStation	0	0

Vehicles and gas station communication	Payment	0.000297561	1.03
	sendMessage	0.000244863	0.85
	readMessage	0	0
	requestService	0.000470134	1.63

**Table 5:** Comparison of proposed system with related work.

	[22]	[23]	[24]	[25]	Our system
Blockchain-based	Yes	Yes	Yes	Yes	Yes
Smart contracts	No	Yes	Yes	No	Yes
Access control-based	No	No	No	No	Yes
Multi-agent system	No	No	No	No	Yes
Security	Yes	Yes	No	Yes	Yes
Trustfulness	No	No	Yes	Yes	Yes

## Data Availability

The data used to support the findings of this study are included within the article.

## References

[1] Almalki F. A., Ben Othman S., Almalki F. A., Sakli H. (2021). Eerp-Dpm: Energy efficient routing protocol using dual prediction model for healthcare using IoT. *Journal of Healthcare Engineering*.

[2] Wu Y., Guo H., Chakraborty C., Khosravi M., Berretti S., Wan S. (2022). Edge computing driven low-light image dynamic enhancement for object detection. *IEEE Transactions on Network Science and Engineering*.

[3] Othman S. B., Almalki F. A., Chakraborty C., Sakli H. (2022). Privacy-preserving aware data aggregation for IoT-based healthcare with green computing technologies. *Computers & Electrical Engineering*.

[4] Hakak S., Gadekallu T. R., Ramu S. P., Maddikunta P. K. R., de Alwis C., Liyanage M. (2022). Autonomous Vehicles in 5G and beyond: A Survey. https://arxiv.org/abs/2207.10510.

[5] Saxena S., Bhushan B., Ahad M. A. (2021). Blockchain based solutions to Secure Iot: background, integration trends and a way forward. *Journal of Network and Computer Applications*.

[6] Bhushan B., Sahoo C., Sinha P., Khamparia A. (2020). Unification of Blockchain and Internet of Things (BIoT): requirements, working model, challenges, and future directions. *Wireless Networks*.

[7] Acharya V., Ravi V., Pham T. D., Chakraborty C. (2021). Peripheral Blood Smear Analysis Using Automated Computer-Aided Diagnosis System to Identify Acute Myeloid Leukemia. *IEEE Transactions on Engineering Management*.

[8] Haque A. K. M. B., Bhushan B., Dhiman G. (2021). Conceptualizing smart city applications: requirements, architecture, security issues, and emerging trends. *Expert Systems*.

[9] Mhamdi H., Zouinkhi A., Sakli H. Smart contracts for decentralized vehicle services.

[10] Mhamdi H., Zouinkhi A., Sakli H. Multi-agents’ system of vehicle services based on Blockchain.

[11] Bhuyan H., Chakraborty D. C., Pani S., Ravi V. (2021). Feature and Subfeature Selection for Classification Using Correlation Coefficient and Fuzzy Model. *IEEE Transactions on Engineering Management*.

[12] Deepa N., Pham Q. V., Nguyen D. C. (2022). A survey on blockchain for big data: approaches, opportunities, and future directions. *Future Generation Computer Systems*.

[13] Sakli N., Ghabri H., Soufiene B. O. (2022). ResNet-50 for 12-lead electrocardiogram automated diagnosis. *Computational Intelligence and Neuroscience*.

[14] Almalki F. A., Soufiene B. O. (2021). EPPDA: an efficient and privacy-preserving data aggregation scheme with authentication and authorization for IoT-based healthcare applications. *Wireless Communications and Mobile Computing*.

[15] Soufiene B. O., Abdullah A. B., Trad A., Youssef H. Peerp: A priority-based energy-efficient routing protocol for reliable data transmission in healthcare using the IoT.

[16] Javed A. R., Hassan M. A., Shahzad F. (2022). Integration of blockchain technology and federated learning in vehicular (IoT) networks: a comprehensive survey. *Sensors*.

[17] Chen C., Wu J., Lin H., Chen W., Zheng Z. (2019). A secure and efficient blockchain-based data trading approach for Internet of vehicles. *IEEE Transactions on Vehicular Technology*.

[18] Liu K., Chen W., Zheng Z., Li Z., Liang W. (2019). A novel debt-credit mechanism for blockchain-based data-trading in Internet of vehicles. *IEEE Internet of Things Journal*.

[19] Yang Z., Yang K., Lei L., Zheng K., Leung V. C. M. (2019). Blockchain based decentralized trust management in vehicular networks. *IEEE Internet of Things Journal*.

[20] Xia S., Lin F., Chen Z., Tang C., Ma Y., Yu X. (2020). A bayesian game based vehicle-to-vehicle electricity trading scheme for blockchain-enabled Internet of vehicles. *IEEE Transactions On Vehicular Technology*.

[21] Javaid U., Aman M. N., Sikdar B. (2020). A scalable protocol for driving trust management in Internet of vehicles with blockchain. *IEEE Internet of Things Journal*.

[22] Vangala A., Bera B., Saha S., Das A. K., Kumar N., Park Y. (2021). Blockchain-enabled certificate-based authentication for vehicle accident detection and notification in intelligent transportation systems. *IEEE Sensors Journal*.

[23] Jabbar R., Fetais N., Kharbeche M., Krichen M., Barkaoui K., Shinoy M. (2021). Blockchain for the Internet of vehicles: how to use blockchain to secure vehicle-to-everything (V2X) communication and payment?. *IEEE Sensors Journal*.

[24] Singh P. K., Singh R., Nandi S. K., Ghafoor K. Z., Rawat D. B., Nandi S. (2021). Blockchain-based adaptive trust management in Internet of vehicles using smart contract. *IEEE Transactions on Intelligent Transportation Systems*.

[25] Zhao Y., Wang Y., Wang P., Yu H. (2022). PBTM: a privacy-preserving announcement protocol with blockchain-based trust management for IoV. *IEEE Systems Journal*.

